# Wunsch nach Suizidassistenz in der spezialisierten ambulanten Palliativversorgung (SAPV) – Eine Fallserie und Implikationen für das Team

**DOI:** 10.1007/s00103-026-04200-2

**Published:** 2026-02-17

**Authors:** Kerstin Kremeike, Heidrun Golla, Ruben Albrecht, Thomas Montag, Angelika Mollidor, Lisa Pusch, Julia Schmidtke, Uta Welling, Raymond Voltz

**Affiliations:** 1 Zentrum für Palliativmedizin, Medizinische Fakultät und Universitätsklinikum Köln, Universität zu Köln, Kerpener Str. 62, 50937 Köln, Deutschland; 2 Klinik für Palliativmedizin, Universitätsmedizin Göttingen, Göttingen, Deutschland; 3 Medizinische Fakultät und Universitätsklinikum, Zentrum für Integrierte Onkologie Aachen Bonn Cologne Düsseldorf (CIO ABCD), Universität zu Köln, Köln, Deutschland; 4 Medizinische Fakultät und Universitätsklinik, Zentrum für Versorgungsforschung Köln (ZVFK), Universität zu Köln, Köln, Deutschland

**Keywords:** Suizidassistenz, Todeswünsche, Spezialisierte ambulante Palliativversorgung, Lebensende, Fallstudie, Assisted suicide, Desire to die, Specialized outpatient palliative care, End of life, Case study

## Abstract

**Einleitung:**

Seit der Aufhebung des § 217 StGB Anfang 2020 stellen sich auch in der spezialisierten ambulanten Palliativversorgung (SAPV) neue Herausforderungen im Umgang mit Wünschen nach Suizidassistenz, die bislang kaum systematisch untersucht wurden. Ziel der Arbeit ist es, Fälle mit einem Wunsch nach Suizidassistenz in der SAPV darzustellen und zu analysieren. Auf dieser Grundlage sollen zentrale Herausforderungen in der Versorgung identifiziert und Ansatzpunkte für Strategien zum Umgang mit diesen Wünschen entwickelt werden.

**Methoden:**

Es wurde eine retrospektive Fallanalyse aller Patient:innen durchgeführt, die zwischen 02/2020 und 08/2025 durch das SAPV-Team der Uniklinik Köln betreut wurden und einen expliziten Wunsch nach Suizidassistenz geäußert haben. Die Daten wurden aus der elektronischen Dokumentation (PalliDoc®) extrahiert. Versorgungspraktische und -ethische Fragestellungen wurden im multiprofessionellen Team abgeleitet.

**Ergebnisse:**

Insgesamt wurden sechs Patient:innen eingeschlossen. Fünf litten an amyotropher Lateralsklerose, eine Person an einer fortgeschrittenen onkologischen Erkrankung. Drei Patient:innen nahmen externe Suizidassistenz in Anspruch.

**Diskussion:**

Zentrale Herausforderungen in der Versorgung betrafen die Rollenklärung der SAPV, die Kommunikation über Suizidassistenz, die Begleitung von Nahestehenden sowie den Versorgungsabschluss. Eine klare organisationsinterne Haltung sowie definierte Kommunikationswege im Team und gegenüber Patient:innen sowie deren Nahestehenden wirken unterstützend. Die Arbeit formuliert praxisrelevante Fragestellungen zur konzeptionellen Weiterentwicklung des Umgangs mit Wünschen nach Suizidassistenz.

## Abstract (en)

**Introduction:**

Following the repeal of § 217 of the German Criminal Code in early 2020, new related challenges have also emerged in specialized outpatient palliative care (SAPV) regarding how to address requests for assisted suicide that have so far received little systematic investigation. The aim of this study is to describe and analyze cases involving requests for assisted suicide within SAPV. On this basis, key challenges in care are identified and potential strategies for dealing with such requests are developed.

**Methods:**

A retrospective case analysis was conducted of all patients who were cared for by the SAPV team at the University Hospital Cologne between February 2020 and August 2025 and explicitly expressed a wish for assisted suicide. Data were extracted from the electronic documentation system (PalliDoc®). Practical and ethical issues related to care were derived within the multiprofessional team.

**Results:**

A total of six patients were included. Five patients suffered from amyotrophic lateral sclerosis and one patient had advanced oncological disease. Three patients made use of external assisted suicide services.

**Discussion:**

Central challenges in care concerned clarification of the role of SAPV, communication about assisted suicide, support of relatives, and the conclusion of care. A clear internal organizational position, as well as defined communication pathways within the team towards patients and their relatives, proved to be supportive. This study formulates practice-relevant questions for the conceptual further development of approaches to requests for assisted suicides.

## Einleitung

Im Februar 2020 erklärte das Bundesverfassungsgericht den § 217 des Strafgesetzbuches (StGB) für nichtig und hob damit das 2015 gesetzlich eingeführte Verbot einer geschäftsmäßigen Förderung der Selbsttötung in Deutschland auf [1]. Auch in der Hospiz- und Palliativversorgung erfährt die Suizidassistenz seither gesteigerte Aufmerksamkeit – sowohl durch Patient:innen und deren Nahestehende als auch seitens der Versorgenden.

Die Hospiz- und Palliativversorgung umfasst ambulante und stationäre Versorgungsstrukturen [2]. Dabei wird jeweils zwischen der allgemeinen und spezialisierten Palliativversorgung unterschieden [3, 4]. Allgemein palliativ versorgt werden Patient:innen mit weniger komplexen Symptomen als integraler Bestandteil der Primär- und Grundversorgung. Eine spezialisierte Palliativversorgung erhalten Menschen mit einer fortschreitenden unheilbaren Erkrankung und komplexen Anforderungen – vorübergehend oder dauerhaft, die durch andere Behandlungsmöglichkeiten nicht hinreichend abgedeckt werden können [5].

Die spezialisierte ambulante Palliativversorgung (SAPV) kooperiert eng mit niedergelassenen Ärzt:innen, ambulanten Pflegediensten, Krankenhäusern und stationären Pflegeeinrichtungen sowie der Hospizarbeit. Gemeinsames Ziel ist, die Lebensqualität und Selbstbestimmung von Patient:innen so weit wie möglich zu fördern [6]. Die SAPV beinhaltet spezialisierte palliativärztliche und -pflegerische Beratung und/oder (Teil‑)Versorgung, einschließlich der Koordination von Versorgungsleistungen sowie einer 24-stündigen Erreichbarkeit in Krisensituationen. Auch die psychosoziale Versorgung spielt in der SAPV eine zentrale Rolle, um Belastungen im häuslichen Umfeld frühzeitig zu erkennen, Patient:innen und Nahestehende individuell zu begleiten und somit Stabilität und Lebensqualität im Alltag zu fördern. Im Bundesrahmenvertrag zur SAPV ist die entsprechende Berufsgruppe jedoch nicht als fester Teil des Teams vorgesehen.

Seit Abschaffung des § 217 StGB nimmt die Anzahl dokumentierter assistierter Suizide in Deutschland kontinuierlich zu: Die drei größten Sterbehilfevereine berichteten 109 begleitete Suizide im Jahr 2020 und 977 im Jahr 2024 [7]. Laut Schätzungen u. a. der Deutschen Gesellschaft für Humanes Sterben (DGHS) als eine dieser Sterbehilfeorganisationen waren es 2024 insgesamt etwa 1200, weil auch Begleitungen durch kleinere Vereine oder einzelne Ärzt:innen erfolgten [7]. Deutschland führt kein amtliches Register für assistierte Suizide, sie werden nicht zentral erfasst und das Statistische Bundesamt weist nur Suizide insgesamt aus.

Bei Weitem nicht alle schwerkranken Menschen, die Anfragen nach Suizidassistenz stellen oder anderweitig Todeswünsche äußern, beenden ihr Leben tatsächlich vorzeitig mit oder ohne Unterstützung Dritter. Das Äußern von Todeswünschen kann auch der Versuch sein, Kontrolle zu behalten sowie existenzielles Leid zum Ausdruck bringen – oder auch einen Lebenswillen [4]. Anfragen nach Suizidassistenz oder anders geäußerte Todeswünsche können mit ganz unterschiedlich starkem Handlungsdruck und dahinterliegenden Motivationen einhergehen. Darauf Einfluss nehmende Faktoren lassen sich der körperlichen (z. B. Symptomlast), psychischen (z. B. Depression, Hoffnungslosigkeit oder wahrgenommene Selbstwirksamkeit), sozialen (z. B. das Gefühl, eine Last zu sein) oder spirituellen Dimension (Wahrnehmung von Würde oder Sinn im Leben) zuordnen [8].

Auch in der SAPV äußern Patient:innen Wünsche nach Suizidassistenz. Durch offene, interessierte und wertfreie Gespräche, Maßnahmen zur Symptomlinderung und Versorgungsverbesserung sowie Informationsvermittlung treten solche Wünsche oft in den Hintergrund [4]. Versorgende benötigen dennoch immer ein strukturiertes Vorgehen, sobald ein entsprechender Wunsch geäußert wird – unabhängig davon, ob er dauerhaft, freiverantwortlich und autonom ist –, also aus eigenem, einsichtsfähigem Antrieb heraus geäußert wird [9]. Für den hospizlichen [10], pflegerischen [11] und hausärztlichen Bereich [12] liegen bereits strukturierte Handlungsempfehlungen vor. Herausforderungen, die mit Wünschen nach Suizidassistenz durch Patient:innen in der SAPV verbunden sind, wurden bisher nicht systematisch untersucht.

Die vorliegende Fallserie stellt Patient:innen vor, die seit Abschaffung des § 217 einen ausdrücklichen Wunsch nach Suizidassistenz gegenüber dem SAPV-Team der Uniklinik Köln geäußert haben. Entsprechend der Position der Bundesärztekammer, die die Mitwirkung bei der Selbsttötung nicht als ärztliche Aufgabe sieht, definiert das SAPV-Team die Suizidassistenz nicht als Teil seines Versorgungsangebots [13]. Trotz einer klaren Haltung in diesem Punkt, wirft die Begleitung von Patient:innen mit einem Wunsch nach Suizidassistenz herausfordernde praxisrelevante Fragen auf. Die vorliegende explorative, unizentrische Fallserie fokussiert die Beschreibung prozessualer Aspekte und die Generierung von Fragen, die SAPV-Teams als Leitfragen für die Entwicklung tragfähiger Strategien zum Umgang mit Anfragen nach Suizidassistenz dienen können.

## Methoden

Untersucht werden die Versorgungsverläufe aller SAPV-Patient:innen der Uniklinik Köln, die im Zeitraum von 02/2020 bis 08/2025 den konkreten Wunsch nach Suizidassistenz geäußert haben. Dies erfolgt in Abgrenzung von regelmäßig auftretenden Todeswünschen, bei denen ein geringerer Handlungsdruck besteht. Diese Todeswünsche werden verstanden als häufig ambivalente (Leidens‑)Aussagen ohne konkreten dauerhaften Planungsbezug. Sie nehmen mit symptomatischer, psychosozialer und spiritueller Linderung oft ab. Demgegenüber ist der Wunsch nach Suizidassistenz eine zielgerichtete Bitte um Mithilfe Dritter – typischerweise mit benannten Schritten wie der Kontaktaufnahme zu durchführenden Organisationen oder Ärzt:innen, der Klärung von Verfahren und der Bereitstellung von tödlichen Substanzen.

In die Fallstudie wurden alle Patient:innen eingeschlossen, die im genannten Zeitraum einen konkreten Wunsch nach Suizidassistenz explizit gegenüber dem SAPV-Team äußerten – unabhängig davon, ob eine Suizidassistenz später tatsächlich durchgeführt wurde. Einschlusskriterium war damit, dass das Thema Suizidassistenz im Behandlungsverlauf erkennbar versorgungsrelevant war und das Team, v. a. kommunikativ, besonders herausforderte. Damit sollen die praktischen und ethischen Spannungsfelder sichtbar werden, aus denen sich die für Versorgungsteams relevanten Fragestellungen in Bezug auf die Suizidassistenz ableiten.

Die fallbezogene Extraktion aller im elektronischen Dokumentationssystem (PalliDoc®) verschriftlichten Informationen erfolgte retrospektiv. Die gesamte fallbezogene Dokumentation wurde im Rahmen eines qualitativ-inhaltsanalytischen Vorgehens vollständig gesichtet. Ausgehend vom Datenmaterial erfolgte die Zusammenfassung themenrelevanter Aspekte so, dass eine Rückidentifizierung der Patient:innen für Außenstehende nicht möglich ist. Die Fallberichte orientieren sich an den CAse REports (CARE) Guidelines [14]. Für die Ableitung versorgungspraktischer und -ethischer Fragestellungen wurden qualitativ-explorativ Herausforderungen des SAPV-Teams bezüglich der Suizidassistenz erhoben. Dazu fanden multiprofessionelle Besprechungen statt, in denen die 6 Fallverläufe gemeinsam reflektiert und protokolliert wurden. Die Erstautorin, die nicht in die Versorgung der Patient:innen eingebunden war, führte zudem problemzentrierte Einzelinterviews mit ausgewählten Teammitgliedern. Aus den wiederkehrenden Themen wurden in einem iterativen Analyseprozess die zusammengefassten Herausforderungen abgeleitet

## Ergebnisse

Von den insgesamt 2049 Patient:innen, die von 02/2020 bis 08/2025 im Rahmen der SAPV der Uniklinik Köln betreut wurden, äußerten sechs den konkreten Wunsch nach Suizidassistenz gegenüber den Versorgenden (0,3 %). Fünf litten an amyotropher Lateralsklerose (ALS), eine:r an einem weit fortgeschrittenen Tumor. Drei der sechs Patient:innen nahmen Suizidassistenz durch Sterbehilfevereine tatsächlich in Anspruch. Ein:e Patient:in verstarb vor Ablauf der vorgegebenen Wartezeit der Sterbehilfeorganisation zu Hause, eine:r aus natürlicher Ursache im Hospiz, ein:e dritte:r wurde gut symptomkontrolliert in eine Pflegeeinrichtung entlassen. Weitere Details zur Versorgungssituation zeigt Tab. 1, Zusammenfassungen der einzelnen Fälle folgen chronologisch.

**Tab. 1:** Merkmale und (Versorgungs‑)Situation der Patient:innen mit Wunsch nach Suizidassistenz in der spezialisierten ambulanten Palliativversorgung (*SAPV*)

Merkmale	Ausprägung	Anzahl (*n*)
Erkrankung	Amyotrophe Lateralsklerose (ALS)	5
	Weit fortgeschrittenes Tumorleiden	1
Alter bei Versorgungsbeginn (Mittel/Median (min.–max.))	68/67 (57–73) Jahre	–
Geschlecht	Männlich	3
	Weiblich	3
Versorgungdauer SAPV (Mittel/Median (min.–max.))	9/6,5 (5–17) Monate	–
Grund für Versorgungsende	Inanspruchnahme von Suizidassistenz durch Sterbehilfeverein	3
	Versterben an der Erkrankung ohne Inanspruchnahme von Suizidassistenz	2
	Entlassung aus der SAPV, da Bedarf nicht mehr gegeben	1
(Mit‑)Versorgung	Durch Partner:in	5
	Pflegedienst, Haus- und Fachärzt:innen	6
	Stationäres Hospiz	1
Häufigste leidvolle Symptome sowie Herausforderungen, die die Entscheidung für die Suizidassistenz begünstigten	Empfundener Autonomieverlust durch zunehmende Schwäche und/oder Immobilität	6
	Belastete Angehörige	5
	Schmerzen	5
	Stationäre Unterbringung nur mit Hindernissen zu organisieren (z. B. durch eingeschränkte Kommunikationsfähigkeit der Patient:innen)	3

### Fall 1 – „Nicht bis zum Endstadium“

Patient:in mit familiärer ALS wurde zu Hause von Partner:in, unterstützt durch eine Familienangehörige sowie einen Pflegedienst, versorgt. Zunehmende Kommunikations- und Schluckstörungen sowie der Verlust von Selbstständigkeit und wahrgenommener Würde führten zu anhaltender Belastung und der Sorge, den Nahestehenden zur Last zu fallen. Trotz stabilisierender Symptomtherapie und Hilfsmittelberatung bekräftigte Patient:in den Wunsch, das Endstadium der Erkrankung, dass von einem engen Familienmitglied bereits bekannt war, nicht erleben zu müssen. Das SAPV-Team klärte Möglichkeiten und Grenzen palliativer Versorgung, hielt eine klare Abgrenzung zur Durchführung der Suizidassistenz und blieb für Gespräche und Krisenpläne verfügbar. Wesentliche Inhalte der Gespräche mit dem SAPV-Team waren für Patient:in die konkrete praktische Umsetzung der Suizidassistenz mit einer Sterbehilfeorganisation und das Erfüllen der dafür notwendigen Formalia. Kurz vor einem ohnehin erwartbar nahen natürlichen Sterben nahm Patient:in Suizidassistenz über eine externe Organisation in Anspruch; die Entlassung aus der SAPV erfolgte kurz zuvor, das Team blieb für die Nahestehenden nach dem Ereignis ansprechbar. Diese sprachen ihre Zufriedenheit darüber aus, dass der Wunsch des/der Patient:in bezüglich der Gestaltung des Lebensendes erfüllt worden war.

### Fall 2 – „Genussverlust und Abhängigkeit“

Patient:in mit ALS lebte mit Partner:in, welche:r die häusliche Versorgung maßgeblich trug. Besonders einschneidend war für Patient:in der körperliche Verfall und hier vor allem – aufgrund einer Schluckstörung – die abnehmende Fähigkeit zu gemeinsamen Mahlzeiten, die für Patient:in den Verlust von Lebensqualität und Autonomie symbolisierte. Unter regelmäßigen Kontakten mit dem SAPV-Team wurden Schmerz, Angst und Schlaf verbessert und ein Versorgungsplan erstellt. Patient:in äußerte bereits bei Kontaktaufnahme zum SAPV-Team den grundsätzlichen Wunsch nach Suizidassistenz und verwies dabei auf den durch das Urteil des Bundeverfassungsgerichts aus dem Jahr 2020 bestehenden Anspruchs darauf („Sie müssen das doch jetzt machen“). Nach Aufklärung darüber, dass Suizidassistenz durch das SAPV-Team nicht durchgeführt werde und alternative Behandlungsangebote bestünden, wurden diese gern in Anspruch genommen. Patient:in gab an, insbesondere von den regelmäßigen Gesprächen zu profitieren. Später registrierte sich Patient:in bei einem Sterbehilfeverein, empfand jedoch Wartezeiten und organisatorische Hürden als belastend. Bei rascher Verschlechterung der ALS verstarb sie/er vor Umsetzung der Suizidassistenz unter guter Symptomkontrolle zu Hause, Partner:in beschrieb Trauer, aber auch Erleichterung über das friedliche Sterben.

### Fall 3 – „Ambivalenz und Alternativen“

Alleinlebende:r Patient:in mit ALS verfügte über ein breites Unterstützungsnetz. Fortschreitende Paresen, Atemnot und Schmerzen führten zu starkem Autonomieverlust. Trotz intensiven Komplexsymptom-Managements im häuslichen Setting äußerte Patient:in immer wieder den Wunsch nach assistierter Lebensbeendigung, lehnte aber kommerzielle Angebote ab und engagierte sich öffentlich für Zugangsrechte. Das SAPV-Team wurde wiederholt um Rezepte für Mittel gebeten, die im Sinne einer Selbsttötung eingenommen werden könnten. Daraus entwickelten sich regelmäßig ausführliche Gespräche über den Wunsch zu sterben, diesbezügliche Möglichkeiten und Handlungsspielräume. Es kam zu einem nicht konsequenten Suizidversuch und mehreren Klinikkontakten. Nach einem psychiatrischen Konsil, das Patient:in keine Psychopathologie zusprach, kam es zu einem vorübergehenden freiwilligen Verzicht auf Essen und Trinken. Dieser wurde später beendet und Patient:in stabilisierte sich. Nach einer Hospizphase erfolgte die Verlegung in eine Pflegeeinrichtung ohne weitere SAPV-Begleitung.

### Fall 4 – „Rote Linie Inkontinenz und Bettlägerigkeit“

Patient:in mit fortgeschrittener onkologischer Erkrankung wurde zu Hause durch Partner:in und einen Pflegedienst versorgt. Inkontinenz und Immobilität markierten eine persönliche Grenze in Bezug auf Würde und Selbstbestimmung. Das SAPV-Team leistete kontinuierliche, strukturierte Symptomkontrolle, Gesprächs- und Entscheidungsunterstützung. Die Freiverantwortlichkeit der Entscheidung für die Suizidassistenz erschien den Versorgenden und der Familie unstrittig. In den Gesprächen im Rahmen der SAPV war ein wichtiges Thema für Patient:in, wie der „richtige“ Zeitpunkt für assistierten Suizid zu bestimmen sei. Patient:in war erwartbar nah an einem natürlichen Sterben und wollte durch Inanspruchnahme der Suizidassistenz den eigenen Tod(eszeitpunkt) selbst bestimmen. Alternative Angebote abschirmender Medikation zur Leidlinderung am Lebensende wurden abgelehnt. Die Suizidassistenz fand im häuslichen Umfeld über eine externe Organisation statt. Die Entlassung aus der SAPV erfolgte nach ausführlichem Gespräch und Beratung, kurz bevor der assistierte Suizid vollzogen wurde. Im Anschluss entstanden formale Nachverfahren, die die nahestehende Person belasteten; das SAPV-Team blieb zur Nachsorge und Entlastung im Kontakt.

### Fall 5 – „Lebensende gestalten – Fokus Nahestehende“

Patient:in mit ALS, war bereits bei Beginn der SAPV vollständig pflegeabhängig (u. a. perkutane endoskopische Gastrostomie – PEG, Kommunikation über Hilfsmittel), im Verlauf wurden Mobilisierung und Lagewechsel immer schwieriger. Auch Partner:in war hoch belastet; eine stationäre Entlastung lehnten beide aber aufgrund für sie unpassender Rahmenbedingungen ab. Zu Beginn der SAPV verwies Patient:in auf den in der Patientenverfügung bewusst gewählten Begriff „Sterbehilfe“. Dieser wurde im weiteren Versorgungsverlauf nicht mehr aufgegriffen. Ein zentrales Anliegen war die Gestaltung des eigenen Lebensendes unter Einbezug der Familie und die Vermeidung weiterer Eskalationen. Ein starker Todeswunsch bestand gleichzeitig mit einer großen Traurigkeit in Bezug auf den damit verbundenen Verlust der Nahestehenden. Das SAPV-Team führte fortlaufend stützende Gespräche und optimierte die Symptome. Möglichkeiten der Therapiebegrenzung (Beendigung von Nahrungs- und Flüssigkeitsgabe über die PEG bei weiterlaufender Symptomlinderung) nutzte der/die Patient:in erst spät im Krankheitsverlauf – nach Verlegung in ein Hospiz und verstarb dort im Kreis der Familie. Dies schien für alle Beteiligten sehr passend gestaltet.

### Fall 6 – „Teamrolle klären, Grenzen wahren“

Patient:in mit rasch progredienter ALS lebte mit Partner:in und wurde später neben der SAPV auch durch einen Pflegedienst versorgt, tat sich jedoch schwer, Unterstützung anzunehmen. Partner:in wirkte davon zunehmend belastet. Hauptthemen für Patient:in waren Autonomieverlust, depressive Verstimmung und Schmerzen. Bereits zu Beginn der SAPV war Patient:in bei einer Sterbehilfeorganisation vorgemerkt und benannte Bettlägerigkeit und Verlust der Armfunktion als persönliches Limit. Der dadurch antizipierte verminderte Kontakt zur Umwelt seien die Grenze zu einem dann nicht mehr lebenswerten Leben. Eine Woche vor der geplanten Suizidassistenz informierten Patient:in und Partner:in das SAPV-Team über den anstehenden Termin. Auf die Bitte, das SAPV-Team möge bei der Durchführung der Suizidassistenz anwesend sein, erläuterte das Team seine professionelle Abgrenzung (keine Begleitung der Durchführung), blieb jedoch vor- und nachher erreichbar. Die Entlassung aus der SAPV erfolgte am Durchführungstag; Termin und Ablauf lagen bei der externen Organisation.

## Diskussion

Zusammenfassend erlebten die Patient:innen mit Wunsch nach und teilweise auch Inanspruchnahme von Suizidassistenz vor allem den Verlust an Autonomie sowie die Überlastung ihrer Nahestehenden als schwer aushaltbar. Die Festlegung des Todeszeitpunkts für die Suizidassistenz kann als Versuch verstanden werden, Kontrolle über das eigene Leben und Sterben zurückzugewinnen und damit als Ausdruck der Patientenautonomie. In einer Befragung von 321 Mitgliedern der Deutschen Gesellschaft für Palliativmedizin (DGP), von denen knapp ein Drittel in der SAPV tätig war, wurde Autonomieverlust als häufigster Grund für Wünsche der Patient:innen nach Suizidassistenz angegeben, gefolgt vom Verlust der Fähigkeit, Aktivitäten des täglichen Lebens nachzugehen, der Würde sowie der Kontrolle über körperliche Funktionen [15]. Diese Ergebnisse decken sich mit den genannten Gründen in vorliegender Fallserie. Hier wurde darüber hinaus auch die Belastung der Nahestehenden als entscheidend für die Patient:innen deutlich. Auch die Inanspruchnahme der Suizidassistenz kann Nahestehende sehr belasten [16]. Ihre psychosoziale Begleitung ist damit in jedem Fall ein wichtiger Teil der SAPV [17].

Die sechs dargestellten Versorgungsverläufe umfassen alle Patient:innen des SAPV-Teams der Uniklinik Köln, die seit Abschaffung des § 217 StGB im Februar 2020 bis August 2025 explizit den Wunsch nach Suizidassistenz äußerten und in drei der sechs Fälle mit Unterstützung von Sterbehilfevereinen auch umsetzten. Diese Fälle stellen nur einen sehr geringen Teil (0,3 %) der SAPV-Versorgungen dar, von denen es an der Uniklinik Köln im betrachteten Zeitraum 2049 gab. Während Sterbehilfevereine einen deutlichen Zuwachs an Mitgliedern und durchgeführten Suizidassistenzen seit 2020 berichten, kann dies seitens des SAPV-Teams der Uniklinik Köln für Anfragen nach Suizidassistenz nicht bestätigt werden. Die Durchführung des assistierten Suizids gehört hier derzeit ausdrücklich nicht zum Angebot der SAPV. Von den in bereits oben genannter Erhebung befragten DGP-Mitgliedern gaben 60 % (189/321) an, zwischen 02/2020 und 01/2024 konkrete Anfragen nach Suizidassistenz erhalten zu haben, bei 81 % (152/189) kam dies 1‑ bis 6‑mal vor, lediglich 4 % aller Befragten (12/321) berichteten mehr als 12 Anfragen [15]. Damit scheint weniger die Häufigkeit von Anfragen nach Suizidassistenz entscheidend für SAPV-Teams als vielmehr die konkreten versorgungspraktischen und -ethischen Fragen, die sich im Umgang mit diesen Anfragen ergeben. Denn trotz des geringen Anteils der Inanspruchnahme von oder Anfragen nach Suizidassistenz an der Gesamtzahl der Patient:innen lösten die beschriebenen Fälle einen großen Reflexionsbedarf im SAPV-Team aus. Dies ist auf die besonderen Herausforderungen in der Begleitung dieser Patient:innen zurückzuführen, von denen die Infobox Beispiele aufführt.

Das SAPV-Team der Uniklinik Köln versorgt Patient:innen bis zur tatsächlichen Inanspruchnahme der Suizidassistenz, grenzt sich davon aber – auch zeitlich – klar ab. Trotz dieser scheinbar klaren Haltung stellen sich vielfältige Fragen zur konkreten Umsetzung der Versorgung von Patient:innen mit Wunsch nach oder Inanspruchnahme von Suizidassistenz, die sich aus den in der Infobox angeführten Herausforderungen ergeben. Für die Entwicklung von Strategien zum Umgang damit können konkrete Situationen durchdacht und Vorgehen dafür definiert werden [10]. Für die pflegerische [11] und hospizliche [10] Praxis wurden bereits Fragen formuliert, die sich Versorgende dazu stellen sollten. Aus den hier beschriebenen Versorgungsverläufen und damit verbundenen Herausforderungen lassen sich versorgungsrelevante Fragen generieren. Diese leiten wir in Form von Leitfragen ab, die für SAPV-Teams in der Begleitung von Patient:innen mit Wunsch nach Suizidassistenz bedeutsam sein können (Tab. 2).

**Tab. 2:** Versorgungspraktische Fragen für SAPV-Teams zum Umgang mit Anfragen nach Suizidassistenz

Themen	Versorgungspraktische Fragen	
Aufklärung, Information und Kommunikation über Suizidassistenz	*1.*	*Welche Reflexionsräume und Unterstützung benötigen Mitarbeitende, um das Versorgungsangebot der SAPV gut vermitteln zu können und für einen guten Umgang mit*
		Wünschen nach und Inanspruchnahme von Suizidassistenz ihrer Patient:innen sowie unterschiedlichen Haltungen zur Inanspruchnahme von Suizidassistenz im Team?
	*2.*	*Welche Informationen werden Patient:innen und ihren Nahestehenden über Suizidassistenz gegeben? Z. B.*
		zur Organisation (Anmeldung, Wartezeiten), zum praktischen Ablauf (dass Komplikationen auftreten können, ärztlicherseits der Tod festgestellt und ein Totenschein mit unnatürlicher Todesursache ausgestellt werden muss, dass daraufhin die Polizei eingeschaltet werden und der Leichnam beschlagnahmt werden kann, dass Nahestehende einen besonderen Betreuungsbedarf haben können), zu bisher gemachten Erfahrungen mit Sterbehilfeorganisationen?
	*3.*	*(Wie intensiv) Kommuniziert das Team mit Sterbehilfeorganisationen?*
Durchführung der Suizidassistenz	*4.*	*Wann endet die SAPV?*
	*5.*	*Sind Mitarbeitende des SAPV-Teams auf Wunsch der Patient:innen oder Nahestehenden*
		bei der Durchführung der Suizidassistenz anwesend oder bei Bedarf ansprechbar?
	*6.*	*Steht das SAPV-Team zur Verfügung*
		für die Behandlung von Komplikationen im Rahmen der Suizidassistenz, für die Todesfeststellung und das Ausstellen des Totenscheins, als Ansprechperson für Nahestehende, die Sterbehilfeorganisation oder die Polizei?
Versorgungsabschluss und Nachsorge nach Suizidassistenz	*7.*	*Wie kann ein guter Abschied von Patient:innen und ihren Nahestehenden gelingen?*
	*8.*	*Welche Unterstützungsbedarfe haben Nahestehende in ihrer Trauer?*
	*9.*	*Was braucht es im Team, um einen guten Abschluss mit der Versorgung zu finden?*

Die in Tab. 2 formulierten Leitfragen ergeben sich unmittelbar aus den beschriebenen Fallverläufen und bündeln wiederkehrende Entscheidungssituationen – etwa den Umgang mit geäußerten Wünschen nach Suizidassistenz, die Klärung der Rolle des SAPV-Teams bei Vorbereitung und Durchführung oder die Gestaltung von Abschied und Nachsorge für Nahestehende.

Das SAPV-Team der Kölner Uniklinik lehnt seine professionelle Anwesenheit bei der Durchführung der Suizidassistenz derzeit ab, eine Nachsorge wird durch das Team regelhaft angeboten. Für Komplikationen im Rahmen der Suizidassistenz, das Ausstellen der Todesbescheinigung und auch die Nachsorge für Nahestehende wird die Zuständigkeit bei den Sterbehilfeorganisationen gesehen. Gleichzeitig ist etwa die durch die Versorgung entstandene Nähe zum SAPV-Team häufig auch in der Nachsorge eine wichtige Ressource für Nahestehende. Eine klare Abgrenzung ist hier daher schwierig, aber auch, dass die Nachsorge im Rahmen der SAPV nicht vergütet wird.

Die vorgestellten Fälle veranschaulichen die besondere Relevanz der psychosozialen Begleitung in der SAPV, da Patient:innen und ihre Nahestehenden häufig stark emotional und sozial belastet sind. Obwohl etwa Psycholog:innen, Sozialarbeiter:innen und Seelsorgende maßgeblich zur Entlastung – z. B. bei Ängsten, Trauer oder sozialrechtlichen Fragen – beitragen, sind ihre Leistungen im aktuellen SAPV-Rahmenvertrag für Erwachsene nicht verpflichtend enthalten oder regelmäßig finanziert. Diese Versorgungslücke steht dem Anspruch einer ganzheitlichen Palliativversorgung entgegen und sollte – auch im Hinblick auf eine bestmögliche Suizidprävention – geschlossen werden.

Die in unserem Team erlebten emotionalen Spannungen spiegeln sich auch in der internationalen Literatur wider. Eine systematische Übersicht mit Metasynthese untersuchte emotionale Auswirkungen von „medical aid in dying“ (MAiD) auf beteiligte Gesundheitsfachkräfte [18]. Berichtet wurden sowohl belastende (z. B. Anspannung, Ambivalenz) als auch positive Emotionen (z. B. Erleichterung, Dankbarkeit), insbesondere wenn MAiD als sinnstiftend erlebt wurde. „Moral distress“ entstand u. a. aus Wertekonflikten. In restriktiven Rechtskontexten (z. B. USA) dominierten moralische Belastungen, in offeneren (z. B. Benelux, Schweiz, Kanada) eher reflexive, sinnorientierte Emotionen [18]. Berufsrollen differierten: Pflegefachpersonen beschrieben häufiger emotionale Belastung und Sinnhaftigkeit, Ärzt:innen eher moralische Ambivalenz aufgrund ihres Rollenverständnisses („nicht schaden“ [19]). Die Inanspruchnahme von Suizidassistenz kann also bei Versorgenden ein breites, kontextabhängiges Emotionsspektrum auslösen, das während und nach der Versorgung kontinuierlich reflektiert und fachlich begleitet werden sollte.

Die Auswirkungen nichtassistierter („klassischer“) Suizide auf Versorgende sind psychologisch, sozial, normativ und praktisch deutlich anders gelagert als bei Suizidassistenz (z. B. Unvorhersehbarkeit, traumatische Exposition, Zuschreibungen von Ohnmacht/Schuld). Entsprechend sind emotionale Befunde aus diesem Feld nicht direkt übertragbar. Dennoch lassen systematische Übersichten zu klassischen Suiziden vermuten, dass bestimmte fürsorge- und teambezogene Prozessbausteine auch im Kontext der Suizidassistenz hilfreich sind, z. B. strukturierte Fallbesprechungen, Supervision, klare Kommunikations- und Dokumentationspfade, Postventions‑/Debriefing-Routinen sowie niedrigschwellige Peer-Unterstützung [20, 21]. Darauf aufbauend empfehlen wir für die SAPV bezüglich der Suizidassistenz die präventive Etablierung solcher Formate (z. B. Zugang zu Supervision, Rollen- und Aufgabenklärung sowie ein strukturiertes Vorgehen im Versorgungsteam nach Inanspruchnahme von Suizidassistenz durch Patient:innen), um Belastung zu reduzieren und Handlungsfähigkeit zu sichern. In diesem Rahmen können auch ethisch-normative Entscheidungen selbstkritisch hinterfragt und ggf. angepasst werden, um eine bestmögliche Versorgung sicherzustellen.

Die vorliegende Arbeit stellt reale SAPV-Verläufe von Patient:innen mit konkretem Wunsch nach und teilweise auch Inanspruchnahme von Suizidassistenz dar und erschließt damit praxisnah ein bisher wenig beleuchtetes Themenfeld. Daraus lassen sich relevante Fragen für die Versorgung ableiten. Die Aussagekraft ist durch die kleine Fallzahl sowie das unizentrische und retrospektive Design mit aktenbasierter Datengrundlage begrenzt; damit sind institutioneller sowie Selektions- und Dokumentationsbias möglich, Prävalenzen nicht ableitbar und die Übertragbarkeit ist nur im Sinne analytischer Generalisierung gegeben. Vor diesem Hintergrund verstehen sich die in Tab. 2 formulierten Leitfragen ausdrücklich nicht als abschließende Handlungsempfehlungen, sondern als Ausgangspunkt für weitere Konzeptentwicklung und Forschung. Dafür bietet die Studie wertvolle Impulse. Zur Suizidassistenz in palliativen Versorgungsstrukturen sind multizentrische, prospektive Studien mit einheitlichem Minimaldatensatz nötig, um externe Validität und Vergleichbarkeit zu stärken. Langfristig empfiehlt sich ein datenschutzkonformes Qualitätsmonitoring bzw. ein Register zur Suizidassistenz, um Entwicklungen systematisch abzubilden.

## Fazit

Die Fallstudie zeigt, dass Wünsche nach und die Inanspruchnahme von Suizidassistenz unter Patient:innen in der SAPV selten, aber hochkomplex sind. Sie erfordern eine interessierte und wertfreie Kommunikation, eine offene, aber klare Haltung, auch im Team, und die sensible Begleitung der Patient:innen und ihrer Nahestehenden. Dazu, wie diese Begleitung für alle Beteiligten bestmöglich gestaltet werden kann, stellen sich trotz einer klaren Abgrenzung gegenüber der Durchführung der Suizidassistenz vielfältige Fragen. Kontinuierliche Teamreflexion und strukturelle Standards können Versorgende bei ihrer Beantwortung stärken und auf zukünftige Entwicklungen vorbereiten.

### Infobox Im Team wahrgenommene Herausforderungen bei der spezialisierten ambulanten Palliativversorgung (SAPV) von Patient:innen mit Wunsch nach und/oder Inanspruchnahme von Suizidassistenz

- Wiederholte Notwendigkeit der Klarstellung des Versorgungsangebots der SAPV und diesbezüglicher Grenzen
- Möglichst neutrale Information über Suizidassistenz ohne Beeinflussung der Entscheidung dafür oder dagegen bzw., ohne sich als Versorgende:r oder Team mehr als gewollt zu involvieren
- Bestmögliche palliative, der Symptomlast entsprechende Behandlung und Begleitung des natürlichen Krankheitsverlaufs, auch wenn Patient:innen planen, Suizidassistenz in Anspruch zu nehmen
- Entlassungs- und Übergangsmanagement bei geplanter Suizidassistenz
- Wünsche nach Suizidassistenz nicht als narzisstische Kränkung oder persönliches Versagen zu erleben, da trotz intensiver Bemühungen das Gefühl entstehen kann, nicht ausreichend zu helfen;
- Die Grenzen der Einflussmöglichkeiten auf Patient:innenentscheidungen zu akzeptieren
- Unterschiedliche Haltungen und Belastungen im Team

## Data Availability

Die während der vorliegenden Studie erzeugten und analysierten Datensätze sind auf begründete Anfrage bei der Korrespondenzperson erhältlich.

## References

[1] Bundesverfassungsgericht (2020) Urteil des Zweiten Senats vom 26. Februar 2020. 2 BvR 2347/15, Rn. 1–343.

[2] Kremeike K, Bausewein C, Freytag A et al (2022) DNVF-Memorandum Versorgungsforschung im letzten Lebensjahr. Gesundheitswesen. 10.1055/a-1889-4705 36220106 10.1055/a-1889-4705

[3] Grant M, de Graaf E, Teunissen S (2021) A systematic review of classification systems to determine complexity of patient care needs in palliative care. Palliat Med 35(4):636–650 33706600 10.1177/0269216321996983 PMC8022082

[4] Leitlinienprogramm Onkologie (2020) Palliativmedizin für Patienten mit einer nicht-heilbaren Krebserkrankung – Langversion 2.2. AWMF-Registernummer: 128/001OL 2020. https://www.awmf.org/uploads/tx_szleitlinien/128-001OLk_S3_Palliativmedizin_2020-09_02.pdf. Accessed 16.9.2025

[5] Deutsche Gesellschaft für Palliativmedizin (DGP) (2016) Definitionen zur Hospiz- und Palliativversorgung. https://www.dgpalliativmedizin.de/images/DGP_GLOSSAR.pdf. Accessed 16.9.2025

[6] DGP (2018) Spezialisierte ambulante Palliativversorgung (SAPV). https://www.dgpalliativmedizin.de/allgemein/sapv.html. Accessed 16.9.2025

[7] Forschungsgruppe Weltanschauungen in Deutschland (fowid) (2025) Freitodbegleitungen in Deutschland 2020–2024. https://fowid.de/meldung/freitodbegleitungen-deutschland-2020-2024. Accessed 16.9.2025

[8] Rodríguez-Prat A, Pergolizzi D, Crespo I et al (2024) The wish to hasten death in patients with life-limiting conditions: a systematic overview. J Pain Symptom Manage 68(2):e91–e115. 10.1016/j.jpainsymman.2024.04.023 38703862 10.1016/j.jpainsymman.2024.04.023

[9] Voltz R (2020) Nach Abschaffung des Paragraphen § 217. Wo stehen wir? – Die „Zwei-Hände-Methode“. Z Palliat Med 21(6):288–291

[10] Deutscher Hospiz- und PalliativVerband e. V. (2021) Dialogpapier Hospizliche Haltung in Grenzsituationen. Inhaltliche und methodische Anregungen zur Diskussion und zur Meinungsbildung vor dem Hintergrund des Urteils des Bundesverfassungsgerichts vom 26.02.2020 zu § 217 StGB. https://www.dhpv.de/files/public/stellungnahmen/2021_Dialogpapier-DHPV-EF.pdf. Zugegriffen am 16. September 2025.

[11] Ethikkommission für Berufe in der Pflege Niedersachsen (2024) Konfrontation von Pflege(fach)personen mit Todeswünschen oder Bitten um assistierten Suizid – Wahrnehmungs- und Verhaltenshilfen. https://www.pflegeethikkommission-nds.de/wp-content/uploads/2024/08/Empfehlung_PEK-NDS_2024_1-Lang.pdf. Accessed 16.9.2025

[12] Deutsche Gesellschaft für Allgemeinmedizin und Familienmedizin e. V. (DEGAM) (2024) S1-Leitlinie Umgang mit dem Wunsch nach Suizidassistenz in der hausärztlichen Praxis. https://www.degam.de/files/Inhalte/Leitlinien-Inhalte/Dokumente/DEGAM-S1-Handlungsempfehlung/053-063-umgang-mit-dem-wunsch-nach-suizidassistenz-in-der-hausaerztlichen-praxis/oeffentlich/degam-ll-suizidassistenz-s1_130924.pdf. Accessed 16.9.2025

[13] Bundesärztekammer (2021) Hinweise der Bundesärztekammer zum ärztlichen Umgang mit Suizidalität und Todeswünschen nach dem Urteil des Bundesverfassungsgerichts zu § 217 StGB. Dtsch Arztebl Int 118(29–30):1428–1432

[14] Gagnier JJ, Kienle G, Altman DG et al (2013) The CARE guidelines: consensus-based clinical case reporting guideline development. Glob Adv Health Med 2(5):38–43. 10.7453/gahmj.2013.008 24416692 10.7453/gahmj.2013.008 PMC3833570

[15] Turiaux J, Burner-Fritsch IS, Fegg M, Marckmann G, Bausewein C (2025) Anfragen zu und Praxis von Suizidassistenz – Ergebnisse einer Befragung unter Mitgliedern der Deutschen Gesellschaft für Palliativmedizin (DGP). Bundesgesundheitsblatt Gesundheitsforschung Gesundheitsschutz. 10.1007/s00103-025-04087-5 10.1007/s00103-025-04087-5 PMC12956932 40569373

[16] Wagner B, Hofmann L (2025) Psychosoziale Belastungen von An- und Zugehörigen im Kontext des assistierten Suizids: Ergebnisse einer spezialisierten Beratungsstelle. Gesundheitswesen. 10.1055/a-2633-6971 40744060 10.1055/a-2633-6971

[17] Lin CP, Chu WM (2025) Home-based palliative care: benefits, challenges, opportunities and future directions in a super-aged society. J Hosp Palliat Care 28(3):81–88. 10.14475/jhpc.2025.28.3.81 40919380 10.14475/jhpc.2025.28.3.81 PMC12409080

[18] Dholakia SY, Bagheri A, Simpson A (2022) Emotional impact on staff of end-of-life decisions for children. BMJ Open 12(7):e058523. 10.1136/bmjopen-2021-058523 35840304 10.1136/bmjopen-2021-058523 PMC9295670

[19] Parsa-Parsi RW (2017) The Revised Declaration of Geneva: A Modern-Day Physician’s Pledge. Jama 318(20):1971–1972 29049507 10.1001/jama.2017.16230

[20] Jupina M, Mercer M, Weleff J et al (2024) Prevalence of Patient Suicide and Its Impact on Health Care Professionals: A Systematic Review. Psychiatr Serv 75(10):999–1008. 10.1176/appi.ps.20230351 39350634 10.1176/appi.ps.20230351

[21] Royal College of Psychiatrists (2022) Supporting mental health staff following the death of a patient by suicide: A prevention and postvention framework. https://www.rcpsych.ac.uk/docs/default-source/improving-care/better-mh-policy/college-reports/college-report-cr234-staff-support-following-patient-suicide.pdf. Accessed 16.9.2025

